# Interplay of gut microbiota, immune cells and natural products in reshaping cancer immunotherapy

**DOI:** 10.3389/fmicb.2026.1898063

**Published:** 2026-09-01

**Authors:** Xiaomei Wu, Lingeng Lu, Tingdun Li, Liangyu Zhao, Linlin Wang, Yi Yao, Jian Chen, Tingting Liu, Xiangqian Li, Xiaokang Zhu, Quhuan Ma, Xiaoqin Li, Gongjian Zhu

**Affiliations:** 1 Gansu Provincial Academy of Medical Science, Sun Yat-sen University Cancer Center Gansu Hospital, Lanzhou, China; 2 Yale School of Public Health, New Haven, CT, United States; 3 School of Public Health, Gansu University of Chinese Medicine, Lanzhou, China

**Keywords:** cancer immunotherapy, cytokines, gut microbiota, metabolites, natural products

## Abstract

Recent advances have underscored the gut microbiota as an essential orchestrator of host responses to cancer immunotherapy. Gut microbiota and their metabolites play a vital role in modulating host immune responses. They regulate the development, differentiation, and function of a variety of immune cells, including granulocytes, monocytes, macrophages, dendritic cells (DCs), natural killer (NK) cells, innate lymphoid cells (ILCs), cytotoxic T cells (CTLs), helper T (Th) cells, regulatory T cells (Tregs), and B cells, as well as the production of cytokines such as interleukins (ILs), interferons (IFNs), tumor necrosis factors (TNFs), colony-stimulating factors (CSFs), chemokines, and growth factors (GFs). These cells and molecules are key mediators in shaping immune system. Natural products from meals, herbs, and microbial, plant, or fungal sources act as powerful modulators of gut microbiota. They can reshape microbial composition, regulate microbial metabolites and maintain intestinal barrier integrity. However, the interaction of natural products, gut microbiota, cytokines, and immune cells in modifying cancer immunotherapy is highly complex and remains largely unexplored. In this review, we summarize recent advances in understanding how natural products, microbiota, cytokines, and immune cells orchestrate to reshape the tumor microenvironment, offering novel insights and therapeutic opportunities for improving cancer immunotherapy

## 1 Introduction

Cancers are the second leading cause of death worldwide. Although conventional treatments such as chemotherapy, radiotherapy, and surgery have improved patient outcomes, their effectiveness remains limited, and cancer continues to pose a major global public health challenge due to its high rates of recurrence, drug resistance, and adverse side effects (Bray et al., 2024). Immune escape is a hallmark of malignancies. Many events are involved in immune evasion of human cancers, for example, poor antigen presentation, inhibitory immune checkpoints, and immunosuppressive microenvironment (Wang et al., 2025).

In recent years, immunotherapy, particularly immune checkpoint blockades (ICBs), which aim to reboost the immune system to eliminate malignant cells, has been a breakthrough landmark in cancer treatment (Rui et al., 2023). ICB therapies targeting PD-1/PD-L1 or combined with CTLA-4 inhibition have achieved significant success in melanoma (MM), renal cell carcinoma (RCC), hepatocellular carcinoma (HCC), bladder cancer (BCa), and lung cancer (LC) by reversing T-cell exhaustion and reinvigorating antitumor immunity (Garon et al., 2015; Ott et al., 2019; Linderman et al., 2025). However, “cold tumors,” which are characterized by poor infiltration of cytotoxic immune cells, compromise the efficacy of ICBs. Expanding the therapeutic reach of immunotherapy, therefore, requires urgent exploration of strategies to enhance response rates and overcome resistance mechanisms.

The gut microbiota has recently gained more attention as a key modulator of tumor immunotherapy. Accumulating evidence suggests that these microbial communities enhance therapeutic efficacy by shaping systemic and tumor-specific immune responses (Helmink et al., 2019; Cao et al., 2025; Xie et al., 2025; Lei et al., 2025; Sun et al., 2025). Given its importance, current advanced research has focused on developing therapeutic approaches that target the gut microbiota. For instance, phase I–II clinical trials have demonstrated that fecal microbiome transplantation (FMT) from ICB responders to nonresponders sensitized nonresponders anti-PD-1 therapy in patients with melanoma (Baruch et al., 2021; Routy et al., 2023). Moreover, modulating the gut microbiota through FMT can also reduce immunotherapy-related side effects (Wang et al., 2018).

Besides FMT, other microbiota-modulating approaches such as dietary interventions, probiotics, and natural products have drawn growing interest. In particular, natural compounds are emerging as promising agents that can beneficially remodel the gut microbiota and potentiate antitumor immune responses (Wu and Tan, 2019; Yang et al., 2025; Balaji et al., 2025). These bioactive small molecules or secondary metabolites have long been integral to traditional medicine, exhibiting diverse pharmacological activities, including antioxidant, anti-inflammatory, antimicrobial, cardioprotective, and anticancer effects (Ağagündüz et al., 2022). Nonetheless, the precise mechanisms of how natural products regulate the immune response to tumors by influencing gut microbiota and enhancing the efficacy of tumor immunotherapy are still under investigation (Cheung et al., 2020). In this review, we explore the interplay and underlying mechanisms between the natural products, gut microbiota, and immune cells in the tumor microenvironment (TME) and the role of natural products in cancer immunotherapy. highlighting their potential as adjunctive strategies in cancer treatment.

## 2 Composition and functions of gut microbiota

The human gut is colonized by trillions of microorganisms comprising bacteria, protozoa, fungi, and viruses, which together form the gut microbiota (Valdes et al., 2018). The intestinal flora is mainly composed of anaerobic bacteria. Over 99% of intestinal bacteria belong to the phyla *Bacillota* (synonym “Firmicutes”), Bacteroidota, Actinomycetota, and Pseudomonadota, with Bacillota and Bacteroidota generally representing the dominant groups. Minor phyla such as Verrucomicrobiota, Spirochaetota, and Deferribacterota are usually present at much lower abundances ( < 1%) (Oren and Garrity, 2021). In addition, Archaea, although not bacteria, constitute a small but functionally important component of the gut microbial community. Based on their relationship with the host, the vast number of intestinal bacteria can be broadly divided into three categories: beneficial bacteria-such as *Bifidobacterium pseudolongum* (*B. pseudolongum*), *Lactobacillus plantarum* (*L. plantarum*) and *Alistipes finegoldii* (*A. finegoldii*); pathogenic bacteria-such as *Staphylococcus aureus* (*S. aureus*), *Porphyromonas gingivalis* (*P. gingivalis*), *Clostridium difficile* (*C. difficile*) and *Clostridium perfringens* (*C. perfringens*); and opportunistic pathogens-such as *Fusobacterium nucleatum* (*F. nucleatum*), *Escherichia coli* (*E. coli*), *Bacteroides fragilis* (*B. fragilis*) and *Acinetobacter baumannii* (*A. baumannii*) (Dieterich et al., 2018). Here, the major groups of intestinal bacteria and their mechanism targets are listed in Table 1. The gut microbiota maintains a dynamic, reciprocal relationship with the mammalian immune system, playing a central role in shaping immune responses and enhancing the host’s resistance to infectious diseases (Jordan and Clarke, 2024). Bacterial constituents exert wide-ranging immunoregulatory effects that support overall host health (Ansaldo et al., 2021; Belkaid and Harrison, 2017). These microbial elements can modulate mucosal immunity by interacting with both innate and adaptive immune cells in the gut. Beyond the intestinal environment, the gut microbiota also plays a pivotal role in shaping the development and function of immune cells throughout the body (Wang et al., 2024b). Additionally, gut microbes can indirectly modulate immunity by triggering secondary signaling cascades in intestinal epithelial cells, stromal cells, or resident immune cells (Barbara et al., 2021). Therefore, the balance of gut microbiome is essential for maintaining overall health (Gentile and Weir, 2018; Clemente et al., 2012). Gut microbiota dysbiosis can promote tumor development and compromise responses to cancer treatments, especially immunotherapy (Park et al., 2022).

**TABLE 1:** Typical gut microbiota and their specific functions in tumor immunotherapy.

Bacterial species	Specific functions	Pathogenic bacteria	Beneficial bacteria	Ref.
*F. nucleatum*	Produces succinate to inhibit cGAS–IFN-β, decreases the production of Th1-CCL5 and CXCL10, and suppresses CD8^+^ T cell activation.	Yes		Jiang et al., 2023
*S. aureus*	Secretes leukocidin to promote CRC metastasis.	Yes		Bhattacharya et al., 2018
*C. mitsuokai*	Generates quinolinic acid to disrupt the gut barrier, promoting HCC by activating the PI3K-AKT.	Yes		Zhang Y. et al., 2025
*P. gingivalis*	Enhances neutrophil elastases, facilitating PDAC progression.	Yes		Tan et al., 2022
*F. nucleatum*	Produces butyrate to downregulate PD-1 expression on CD8^+^ T cells in CRC.		Yes	Wang et al., 2024d
*A. muciniphila*	Strengthens gut barrier and increases CD8^+^ T cell infiltration.		Yes	Ding et al., 2025
*B. pseudolongum*	Expands tissue-resident memory CD8^+^ T cells and improves anti-CTLA-4 efficacy.		Yes	Nan et al., 2025
*L. plantarum*	Enhances CD8^+^ T cell responses and produces IPA to promote T cell stemness and antitumor immunity.		Yes	Zhang et al., 2023
*L. murinus*	Activates intestinal DCs.		Yes	Huang et al., 2016
*B. uniformis*	Correlates with IFN-γ+/ CD8^+^ T cells, triggering antitumor immunity.		Yes	Tanoue et al., 2019
*A. finegoldii*	Activates TLR2–NF-κB in DCs to recruit CXCR6^+^/ CD8^+^ T cells to tumors.		Yes	Wu et al., 2025
*R. intestinalis*	Restores gut barrier and activates CD8^+^ T cells via butyrate–TLR5–NF-κB signaling.		Yes	Kang et al., 2023
*R. gnavus*	Promotes NK cell activation, T cell tumor migration, and enhances anti–PD-1 response.		Yes	Di Luccia et al., 2024
*F. prausnitzii*	Ameliorates ICB-induced adverse events by reducing intestinal toxicity and activating CD8^+^ T cells.		Yes	Gao et al., 2023

### 2.1 Gut microbiota metabolites

Under normal physiological conditions, the gut microbiota is confined to the gastrointestinal lumen by the intestinal barrier, which is composed of epithelial cells, mucus, commensal microbes, immune cells, and secreted antibodies (Vancamelbeke and Vermeire, 2017). To overcome this spatial segregation, gut microbes have developed the strategy of releasing diverse metabolites that mediate communication with host tissues and other microbial residents (Belkaid and Hand, 2014). By employing advanced metabolomic technologies, e.g., ultra-performance liquid chromatography coupled with tandem mass spectrometry (UPLC–MS/MS), numerous metabolites, including short-chain fatty acids (SCFAs) and bile acids (BAs), have been successfully characterized. According to their origin, these metabolites can generally be categorized into three groups: (1) those directly synthesized by microorganisms from dietary components, including SCFAs and tryptophan (Trp) metabolites, such as quinolinic acid, indole-3-propionic acid (IPA), indole-3-aldehyde (I3A), indole-3-acetic acid (IAA), and indole-3-acetaldehyde (IAAld); (2) those originating from host compounds that undergo microbial transformation, including secondary BAs, such as deoxycholic acid (DCA) and lithocholic acid (LCA), produced from primary BAs synthesized by the liver, as well as amino acid–derived metabolites such as trimethylamine (TMA), further oxidized to trimethylamine N-oxide (TMAO) in the liver and microbial neurotransmitter precursors including dopamine, serotonin, and gamma-aminobutyric acid (GABA); (3) those produced *de novo* by gut microbes, including polysaccharide A (PSA), peptidoglycan hydrolase derived antigen A (SagA), and lipopolysaccharide (LPS) (Nicholson et al., 2012; Liu et al., 2022; Postler and Ghosh, 2017). Representative metabolites and their role are summarized in Table 2. Each class exerts distinct physiological functions that bridge microbial activity and host responses. Among all the gut microbiota metabolites, the most extensively studied metabolites are SCFAs, bile acids, and amino acid-derived metabolites.

**TABLE 2:** Typical gut microbiota metabolites and their roles in tumor immunotherapy.

Metabolite	Types	Producers	Key Functions	Ref.
Butyrate	SCFAs	Ruminococcaceae, Lachnospiraceae, Clostridiaceae, and Eubacteriaceae	Enhances NKs or CAR-T function via HDAC inhibition through GPCR-CXCL11 signaling pathways, and boosts granzyme B^+^/IFN-γ^+^ CD8^+^ T cell activity.	Kang et al., 2023; Luu et al., 2021; Coutzac et al., 2020; Zhang Y. et al., 2025
Propionate	SCFAs	*M. massiliensis*	Inhibits HDACs and activates mTOR to boost CD8^+^ T cell–mediated antitumor immunity.	Louis and Flint, 2017
Acetate	SCFAs	*B. vulgatus* *L. edouardi*	Reshapes tumor metabolic pathways and enhances PD-L1 expression and immune evasion through the upregulation of c-Myc.	Wang et al., 2024c,Van den Abbeele et al., 2023
Succinate	SCFAs	*F. nucleatum*	Inhibits CD8^+^ T cell infiltration into TME and blocks the cGAS–IFN-β signaling pathway.	Jiang et al., 2023
Inosine	Purine	*B. pseudolongum*	Activates the A2AR–cAMP–PKA–CREB signaling pathway to upregulate IL12 RB2 expression, thereby promoting Th1 differentiation and enhancing antitumor immunity.	Mager et al., 2020
TMAO	Choline	*Clostridiales*	Stimulates M1 macrophages and CD8^+^ T cells infiltration, increasing IFN-γ and TNF-α production, enhancing antitumor effector.	Wang et al., 2022; Mirji et al., 2022
L-Arg	Arginine	*E. coli* and *B. pseudolongum*	Increases DC proportions and CD8^+^ T cell function in tumor-draining lymph nodes, elevating IFN-γ and enhancing the efficacy of anti-PD-L1 therapy.	He et al., 2017; Nan et al., 2025; Canale et al., 2021
SagA	Peptide	*E. faecium*	Increases tumor-infiltrating CD45^+^ cells and CD8^+^ T cells, promoting innate immunity and improving immunotherapy efficacy.	Griffin et al., 2021
IAA	Indole	*I. bartlettii* *Bifidobacterium*	Serves as ligands for the AhR and activate NF-κB, improving immunotherapy efficacy.	Dodd et al., 2017
IAAld	Indole	*E*. *coli*	Activates the AhR-IL-22 axis, thereby enhancing barrier integrity, maintaining immune homeostasis.	Su et al., 2022
I3A	Indole	*L. reuteri*	Activates AhR and stimulates IFN-γ–producing CD8^+^ T cells to improve ICB efficacy.	Bender et al., 2023
IPA	Indole	*L. johnsonii* *C. sporogenes*	Enhances ICB efficacy by metabolizing Trp to regulate CD8^+^ T cells’ stemness and Treg function.	Jia et al., 2024; Fong et al., 2023
Quinolinic Acid	Kynurenine	*C. mitsuokai*	Disrupts the gut barrier and translocates to the liver to accelerate HCC carcinogenesis by PI3K/AKT pathway.	Zhang Y. et al., 2025
Urolithin A	Polyphenol	*L. fermentum*	Enhances the persistence and effector functions of CD8^+^ T cells and CAR-T cells and activates ERK1/2 signaling to stimulate antitumor T cell responses in lymphoma and LC.	Ma et al., 2024; Hua et al., 2027
Microbial vitamin B5	Vitamins	Bacteroidota	Drives CD8^+^ T cell differentiation into IL-22–producing Tc22 cells and boosts anti-PD-L1 efficacy by acting as a coenzyme A precursor.	Bourgin et al., 2022
LCA	BAs	*Clostridium*	Decreases CXCL16 expression in liver sinusoidal endothelial cells, which inhibited CXCR6^+^ NKT cells recruitment and activation.	Ma et al., 2018

SCFAs, primarily acetate, propionate, and butyrate, are major fermentation products of indigestible carbohydrates by gut microbes, with minor contributions from branched-chain amino acid metabolism. They serve as key energy sources for colonocytes and exert systemic effects by activating specific G protein-coupled receptors (GPCRs) and inhibiting histone deacetylases to modulate immune and inflammatory responses (Feng et al., 2018; Tan et al., 2014).

BAs, synthesized from cholesterol in the liver and further metabolized by gut microbiota, act as crucial signaling molecules linking host metabolism and microbial activity. They regulate diverse physiological processes through nuclear receptors such as farnesoid X receptor (FXR) and pregnane X receptor (PXR), as well as membrane receptors including TGR5 and S1PR2, thereby influencing metabolic and immune homeostasis (Gérard, 2013; Jia et al., 2021). Undigested host proteins and peptides can be metabolized by intestinal microbes into a wide variety of nitrogenous and aromatic compounds, including ammonia, amines, sulfides, and diverse indole derivatives, which interact with host receptors such as aryl hydrocarbon receptor (AhR) to regulate metabolism, immunity, and behavior (Agus et al., 2018).

Amino acid metabolites: microbial metabolism of certain amino acids produces bioactive molecules with systemic effects. For instance, gut microbes convert carnitine into trimethylamine, which is further oxidized in the liver to TMAO and linked to cardiovascular risk, while other pathways generate neurotransmitters and precursors such as dopamine, serotonin, and GABA (Ussher et al., 2013). Quinolinic Acid, produced by *C. mitsuokai*, disrupted the gut barrier and translocated to the liver to accelerate HCC carcinogenesis by the PI3K/AKT pathway (Zhang Y. et al., 2025).

## 3 Gut microbiota–cytokines axis in immune regulation and tumor immunotherapy

**FIGURE 1:**
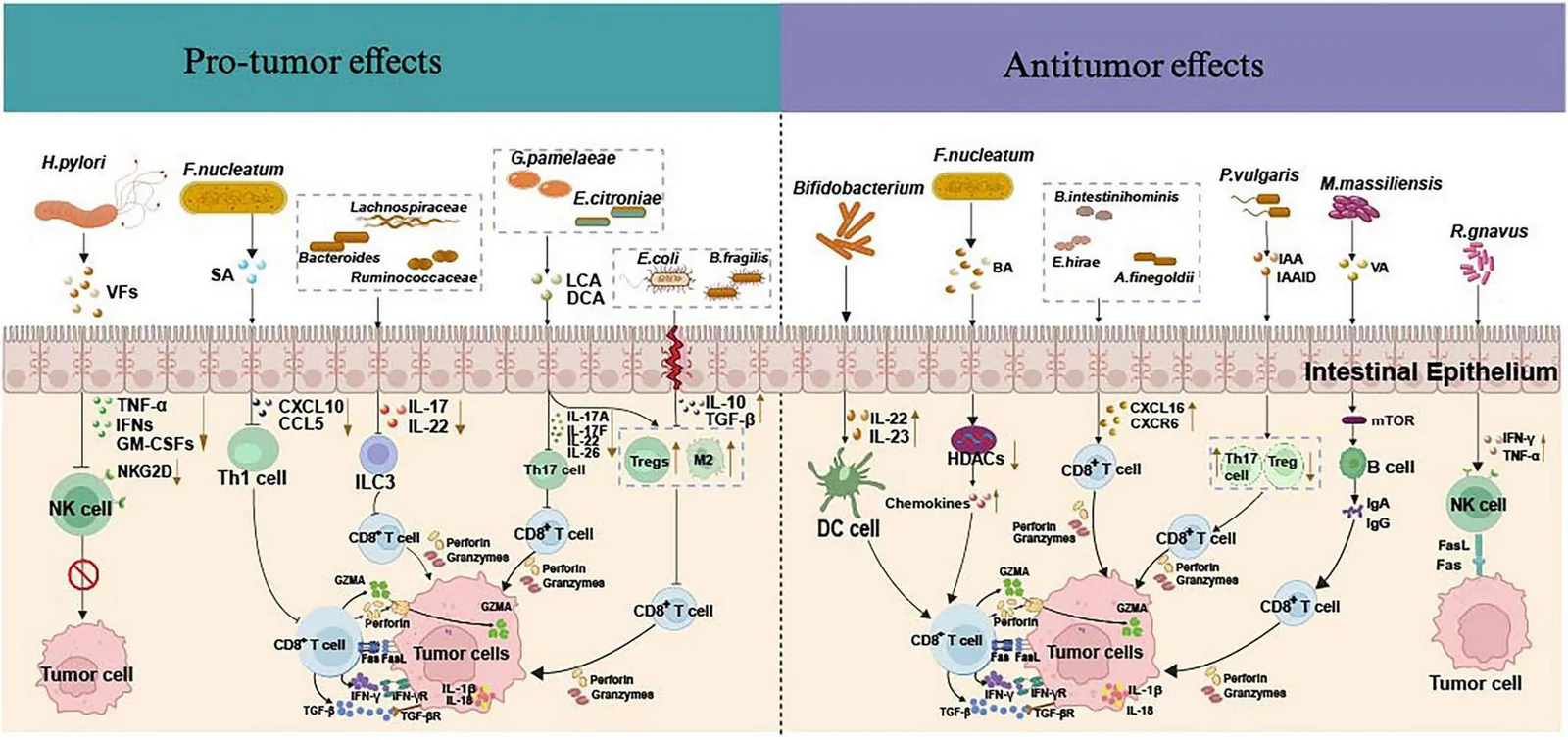
Overview of gut microbiota-cytokine-tumor immunotherapy networks. Gut Microbiota influence tumor immunotherapy through the production of metabolites such as indole-3-acetic acid (IAA) and indole-3-acetaldehyde (IAAld), bile acids (BAs), deoxycholic acid (DCA) and lithocholic acid (LCA), butyric acid (BA), valeric acid (VA), and succinic acid (SA), as well as through virulence factors (VFs). They affect innate immunity by influencing innate immune cells like macrophages (phenotyping M1 and M2), DCs, ILCs and NK cells, and affect adaptive immunity by influencing T cells (Th1, Th17, Tregs, and B cells). Pathogenic bacteria (e.g., *H. pylori*, *F. nucleatum*, *Bacteroides*, Ruminococcaceae, Lachnospiraceae, *G. pamelaeae*, *C. citroniae*, *E. coli*, and *B. fragilis*) drive immunosuppression and enable tumor cells to evade immune surveillance, largely by inducing pro-tumorigenic cytokines such as IL-10 and TGF-β. In contrast, beneficial bacteria, such as *Bifidobacterium*, *E. hirae*, *B. intestinihominis*, *A. finegoldii*, *P. vulgaris*, *Megasphaera massiliensis* (*M. massiliensis*) and *Ruminococcus gnavus* (*R. gnavus*) counteract these effects by strengthening antitumor immunity and promoting the production of anti-tumorigenic cytokines, including IFN-γ, GM-CSFs, TNF-α, IL-17A, IL-17F, IL-22, IL-26, IL-23, CXCL10/CCL5 and CXCL16/CXCR6.

Gut Microbiota and metabolites enhance tumor immunotherapy by shaping host immune system. This system comprises two main arms: innate immunity, which provides a rapid, non-specific response to pathogens, and adaptive immunity, distinguished by its specificity and ability to form immunological memory. It constitutes a critical defense against cancer development (Müller et al., 2017). The gut microbiota engages in continuous bidirectional interactions with immune cells to maintain immune homeostasis. While host immunity regulates microbial composition to preserve intestinal balance, the gut microbiota, in turn, profoundly influences the maturation, activation, and functional programming of immune responses (Fung et al., 2017; Yoo et al., 2020). Cytokines serve as pivotal mediators linking gut microbiota modulation and immune regulation, thus influencing antitumor immune responses and the efficacy of immunotherapy (Propper and Balkwill, 2022; Mendes et al., 2019). The gut microbiota-cytokine-tumor immunotherapy networks are depicted in Figure 1.

### 3.1 Cytokines in tumor immunotherapy

Cytokines are key mediators of cell communication in the TME. They comprise interleukins (ILs), interferons (IFNs), tumor necrosis factors (TNFs), colony-stimulating factors (CSFs), chemokines, and growth factors (GFs) like transforming growth factor-beta (TGF-β), vascular endothelial growth factor (VEGF), and epidermal growth factor (EGF), forming a complex network that allows immune cells to communicate with surrounding cells and tumor cells in the TME. They dramatically affect the many cell types present in tumors and affect cancer outcomes (Dranoff, 2004; Kureshi and Dougan, 2025). Cytokines play a dual role in tumor immunotherapy. Several cytokines, including IFN-α, IFN-γ, CXCL9, CXCL10, and their receptor CXCR3, IL-2, IL-15, IL-10, and granulocyte-macrophage CSF (GM-CSF), can activate and expand CD8^+^ T cells and natural killer (NK) cells and improve the maturation and functions of dendritic cells (DCs), migrating to the tumor and drive antitumor immunity by releasing effector molecules such as granzyme A (GZMA) and perforin (Kureshi and Dougan, 2025). On the other hand, immunosuppressive cytokines like EGF, VEGF, TGF-β, TNF-α, IL-1β, IL-6, IL-12, CSF-1, CCL2, CCL5, and CXCL8 can induce the accumulation of regulatory T cells (Tregs) or M2 macrophages, dampen antitumor immunity, and create an immunosuppressive TME (Derynck et al., 2021). Notably, the net effect of cytokines depends on their type, concentration, and the TME. Some cytokines display context effects on tumor immunity. For example, IL-12, mostly produced by macrophages, can promote antitumor responses by activating Th1 cells and NK cells, yet excessive or prolonged IL-12 signaling may also induce immune exhaustion or tissue inflammation, thereby limiting its therapeutic benefit. mainly boosts activation of Th1 cells (Colombo and Trinchieri, 2002; Mirlekar and Pylayeva-Gupta, 2021). Also, IL-17 is complicated in cancer, functioning as both a promoter of tumor progression and, in specific contexts, a mediator of protective immune responses (Saran et al., 2025; Fabre et al., 2016).

### 3.2 Microbiota-cytokines-driven innate immune modulation

The innate immune system includes physical barriers as well as effector cells like myeloid cells, NK cells, and innate lymphoid cells (ILCs), antimicrobial peptides, soluble mediators, and cell receptors (Aristizábal and González, 2013).

#### 3.2.1 Gut barrier

The gut epithelial barrier, regulated by pattern recognition receptor (PRRs) signaling pathways and specifically by epithelial Toll-like receptor (TLR)-2, acts as the first line of defense against pathogens. The probiotic of Lactobacillus acidophilus (L. acidophilus) reduced TNF-α and protected intestinal epithelial tight junctions by inhibiting NF-κB activation through TLR-2 signaling (Haque et al., 2024). However, certain pathogenic bacteria, such as F. nucleatum, E. coli, and B. fragilis, weaken the intestinal barrier by suppressing epithelial neuropilin-1 (NRP1) and Hedgehog (Hh) signaling through the activation of TLR-2, which disrupts tight junctions, increases intestinal permeability, and sets off inflammatory cascades (Ahmad Kendong et al., 2021; Pontarollo et al., 2023).

#### 3.2.2 Myeloid cells

Myeloid cells, mainly granulocytes, monocytes, macrophages, and DCs, as well as immunoregulatory subsets including myeloid-derived suppressor cells (MDSCs) and M2-polarized tumor-associated macrophages (TAMs), play essential roles in regulating inflammation and maintaining tissue homeostasis. Emerging evidence demonstrates that the gut microbiota critically regulates myeloid cells. For example, Bifidobacterium promotes innate immunity by interacting with DCs, which produce cytokines such as IL-22 and IL-23. This improves the effector function of tumor-specific CD8^+^ T cells, leading to increased IFN-γ production (Sivan et al., 2015). B. fragilis enhanced the phagocytic functions of macrophages, polarizing them to an M1 phenotype and stimulating the release of cytokines such as IL-12 and TNF-α, enhancing antitumor immunity (Deng et al., 2016).

#### 3.2.3 NK cells

NK cells possess intrinsic cytolytic activity that enables them to directly eliminate tumor cells. They also serve as major sources of immunoregulatory molecules, including IFNs, TNF-α, GM-CSFs, and various cytokines and chemokines that modulate immune responses (Campbell and Hasegawa, 2013). Gut microbiota can severely impair the antitumor activity of NK cells, facilitating tumor evasion from immune surveillance. A study reported that the intestinal microbiota facilitated pancreatic ductal adenocarcinoma (PDAC) progression by suppressing intratumoral NK cells activity and reducing IFN-γ expression (Yu et al., 2022). Another study found that *Helicobacter pylori* (*H. pylori*) releases virulence factors (VFs) to reduce the expression of the activation receptor NKG2D on NK cells, hence compromising their activation and cytokine secretion, increasing tumor formation (Sirit and Peek, 2025).

#### 3.2.4 Innate lymphoid cells and cell receptors

ILCs are a class of innate immune cells that exhibit functions reminiscent of adaptive immunity and are categorized into three main groups: ILC1s, ILC2s, and ILC3s. Each subset specializes in the production of distinct cytokines: ILC1s secrete Th1-like cytokines of IFN-γ, ILC2s produce Th2-associated cytokines of IL-5 and IL-13, and ILC3s generate T helper 17 (Th17)/T helper 22 (Th22)-type cytokines, such as IL-17 and IL-22 (Vivier, 2021). Gut pathobionts can alter ILC function, promoting a microenvironment that supports tumor growth. For instance, *Bacteroides*, *Ruminococcaceae*, and *Lachnospiraceae* influence ILC3 dysfunction in colorectal cancer (CRC) patients (Goc et al., 2021).

Gut microbiota also influences innate immune signaling through PRRs. PRRs are a class of receptors that can directly recognize the specific molecular structures on the surface of pathogens, apoptotic host cells, and damaged senescent cells. PRRs bridge nonspecific immunity and specific immunity (Li and Wu, 2021). These microbial components activate PRRs, triggering proinflammatory cytokine production, DC maturation, and NK cell activation, all of which contribute to immune responses and inflammation.

### 3.3 Microbiota-cytokines-driven adaptive immune modulation

Adaptive immunity is mainly mediated by lymphocytes, including T cells and B cells. T cells, consisting of CD4^+^ helper T cells (Th1, Th2, Th17, and Tfh), CD8^+^ cytotoxic T cells (CTLs), and Tregs, regulate or execute cellular immune responses through secreting a subset of cytokines, while B cells mediate humoral immunity by producing antibodies (Chi et al., 2024). Gut microbiota plays an active role in the development and regulation of adaptive immune responses. They can contribute to the development of adaptive immune responses through molecular mimicry in a recognized bacteria-induced immune activation manner, stimulating T cell activation and differentiation, and consequently influencing systemic immune homeostasis (Rossjohn et al., 2025; Garabatos and Santamaria, 2022; Felix et al., 2018). Gut bacteria from Actinomycetota and Bacillota phyla, including *Gordonibacter pamelaeae (G. pamelaeae), Eggerthella lenta (E. lenta)*, *Raoultibacter massiliensis (R. massiliensis)*, *Collinsella intestinalis (C. intestinalis)*, *Adlercreutzia equolifaciens (A. equolifaciens)*, and *Clostridium citroniae (C. citroniae)* produce bile acids that inhibit the differentiation of Th17 cells, influencing the secretion of cytokines such as IL-17A, IL-17F, IL-22, and IL-26 (Paik et al., 2022; Cai et al., 2022). *Clostridium fostered* an environment rich in transforming GF–β to regulate local Tregs accumulation, eliciting antigen-specific T-cell responses (Atarashi et al., 2011). Microbial peptides from Fusobacteria share significant homology with islet self-antigen IGRP, activating IGRP-specific CD8^+^ T cells (Tai et al., 2016). Enterococcus hirae (E. hirae) and Barnesiella intestinihominis (B. intestinihominis) can directly enhance the intratumoral CD8^+^ T cells/Tregs ratio and IFN-γ-producing T cells (Daillère et al., 2016). Additionally, SCFAs generated by gut microbiota boost intestinal IgA and systemic IgG responses by activating mTOR in B cells (Kim et al., 2016).

### 3.4 Microbial metabolites -Cytokines-driven immune regulation

Gut microbiota also influence immune system through their metabolites that diffuse from the gut (Krautkramer et al., 2021). SCFAs, including propionate and butyrate, are crucial microbial metabolites with immunoregulatory properties. Propionate is primarily generated by *Bacteroidota*, *Lachnospiraceae*, and *Roseburia*, whereas butyrate is mainly produced by Bacillota members such as Ruminococcus and Clostridium (Sun et al., 2024). After being absorbed in the colon, SCFAs exert immunomodulatory effects by binding to GPCRs, which regulate cytokine production and cell signaling, by inhibiting histone deacetylases (HDACs), enhancing histone acetylation, and promoting the expression of genes involved in immune regulation (Hinnebusch et al., 2002). Through these mechanisms, SCFAs regulate the differentiation and expansion of Tregs, contribute to the balance of Th1 and Th17 responses, and enhance antitumor immune responses by modulating the activity of macrophages, neutrophils, and DCs (Vinolo et al., 2011; Kim et al., 2014). Secondary BAs, such as DCA and LCA, modulate immune responses partly through their interactions with membrane-bound receptor TGR5 and nuclear receptors such as FXR and PXR (Fiorucci et al., 2018). They regulate T cell differentiation by promoting Treg development while restraining pro-inflammatory Th17 responses, and inducing macrophage polarization to the M2 phenotype, suppressing antitumor immunity (Paik et al., 2022). Indole derivatives, such as IAA and IAAld, synthesized from *E. coli*, *Proteus vulgaris (P. vulgaris)*, *Clostridium spp*., and *Bacteroides spp*., serve as ligands for the AhR (Lee et al., 2015; Tennoune et al., 2022). Upon activation, AhR signaling regulates a broad spectrum of genes, including those encoding CYP1A1, IDO, IL-10, Aiolos, FoxP3, IL-21, and CD39. In T cells, AhR signaling is involved in the regulation of both Th17 and Treg differentiation and consequently influences the Th17/Treg balance and antitumor immunity (Quintana et al., 2008). Collectively, these metabolites serve as key mediators of bidirectional communication between gut microbes and host tissues. They help maintain intestinal barrier integrity and regulate both innate and adaptive immune responses by inducing anti-inflammatory cytokines and chemokines, promoting regulatory T-cell differentiation, and stimulating IgA secretion (Liu et al., 2022).

### 3.5 Dual roles of gut microbiota in tumor immunotherapy

Gut microbiota exerts a complicated and bidirectional influence on tumor immunotherapy. In cancer, an altered gut microbiota enriched with pathogenic bacteria can promote immune evasion and disrupt antitumor immunity, largely by modulating cytokine networks that govern immune cell activation and differentiation. Pathogenic species often induce immunosuppressive cytokines such as IL-10 and TGF-β, leading to impaired cytotoxic T-cell activity and the establishment of a tolerogenic TME. Conversely, beneficial commensal bacteria can restore cytokine homeostasis by enhancing the production of pro-inflammatory cytokines, including IL-12, CXCL9, CXCL10, IFN-γ, and TNF-α, thereby promoting antitumor immune responses. Positive associations were found between the levels of *Lachnoclostridium* and the expressions of CXCL9/10 and CCL5, as well as CD8^+^ T cells in human cancer, including melanoma (Zhu et al., 2021). These commensals have thus emerged as potential probiotics or adjuvants for cancer prevention and therapy through remodeling the tumor microenvironment.

### 3.6 Pathogenic bacteria inhibit antitumor immunity

As pathogenic bacteria, *F. nucleatum* in CRC inhibits the infiltration and function of CD8^+^ T cells, which are essential for recognizing and eliminating tumor cells (Mima et al., 2015). Similarly, *Mycobacterium* spp. can impair T-cell–mediated antitumor responses by promoting regulatory B-cell differentiation and stimulating the secretion of immunosuppressive cytokines, such as IL-10 and TGF-β (Zhou et al., 2017). In addition, during PD-1 and CTLA-4 therapies, recognized carcinogens like *H. pylori* and *Salmonella typhi* (*S*. *typhi*), along with species from the *Bacteroidota*, *Clostridia*, and *Proteobacteria* phyla, have been associated with a higher incidence and severity of immune-related adverse events, highlighting that microbial composition and their metabolites can create an immunosuppressive TME and contribute indirectly to tumor progression (Wizenty and Sigal, 2025; Upadhayay et al., 2022). Collectively, these findings demonstrate that the gut microbiota can profoundly inhibit antitumor immune responses and negatively impact cancer immunotherapy outcomes.

### 3.7 Beneficial bacteria induce antitumor immunity

On the beneficial side, gut microbiota augments immunotherapy efficacy by modulating various aspects of the anti-tumor immune response. In general, commensal bacteria such as *Bifidobacterium bifidum* (*B. bifidum*) and *Akkermansia* spp. enhance dendritic cell activation and CD8^+^ T cells function, improving antigen presentation, stimulating tumor-reactive antibodies, and augmenting cytotoxic T-cell activity (Routy et al., 2018). For example, administration of *B. bifidum* in combination with anti-PD-1 antibodies increased the expression of IFN-α in various immune cells, prompting the accumulation of CD8+ T cells in TME (Lee et al., 2021). *A. finegoldii* enhances chemotactic migration of CD8^+^ T cells via the CXCL16-CXCR6 axis, enhancing immunotherapy efficacy (Wu et al., 2025). *Hominenteromicrobium* (designated YB328) enhances antitumor immunity by accelerating the maturation and migration of DCs to increase the number of CD8^+^ T cells that respond to diverse tumor antigens (Lin et al., 2025). *Akkermansia massiliensis* (*A. massiliensis*) increased CAR-T cell infiltration into bone marrow, inverted the CD4/CD8 CAR-T ratio, favored Tc1 CD8^+^ T cell polarization, and promoted release of Trp-derived indole metabolites, leading to better tumor control (Marcos-Kovandzic et al., 2025). *Lachnoclostridium* is positively associated with CD8^+^ T cells, CXCL9, CXCL10, CXCR5, and better survival in melanoma (Zhu et al., 2021). Collectively, these findings highlight that specific gut microbes can reinforce immune system’s ability to potentiate the effects of immunotherapy.

## 4 Natural products regulate gut microbiota to enhance tumor immunotherapy

**FIGURE 2:**
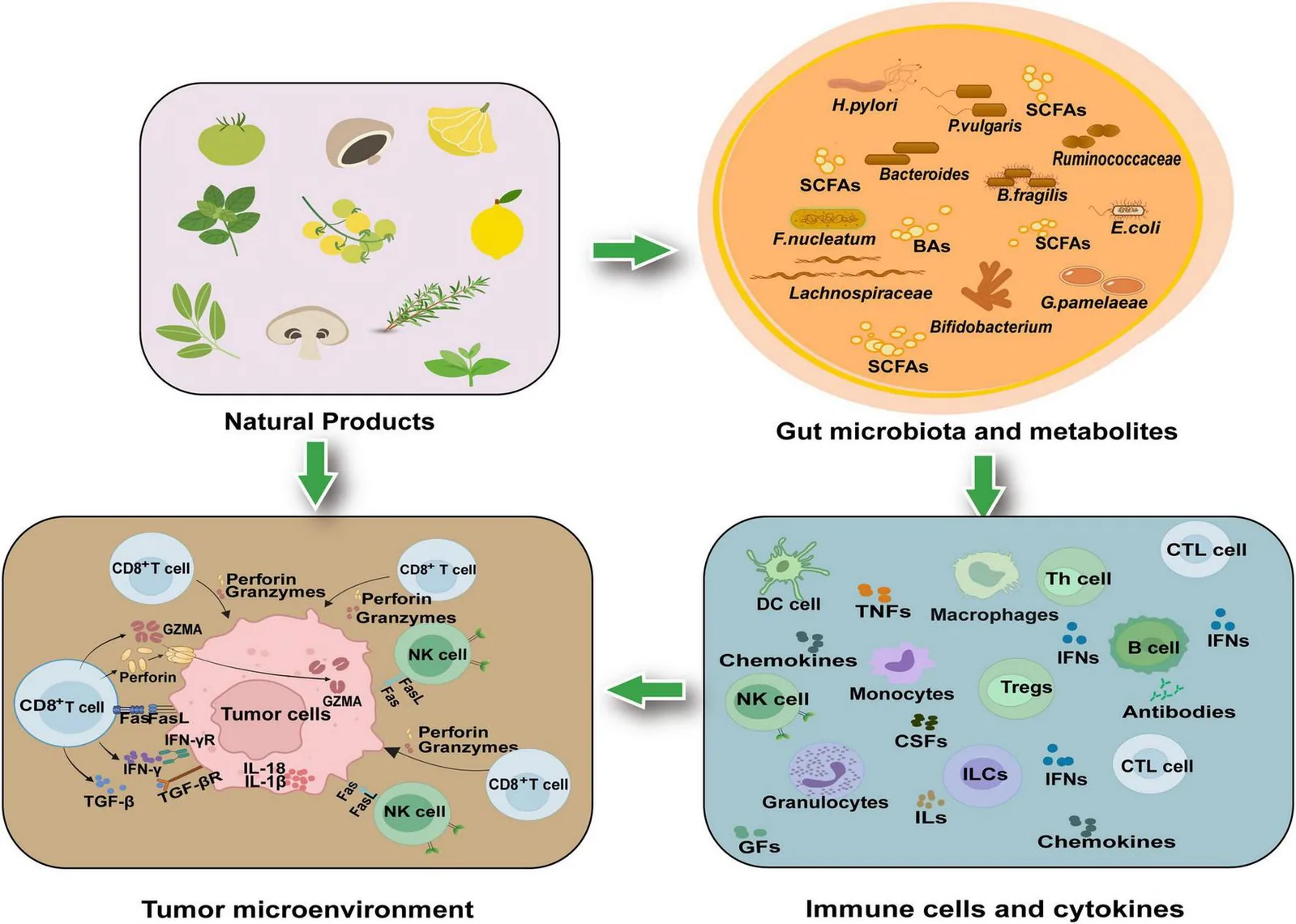
The natural products–gut microbiota–immune axis in cancer immunotherapy.

Natural products, commonly found in foods, spices, herbs, and microbial metabolites, have been recognized for their health-promoting properties. Typically defined as small molecules or secondary metabolites produced by living organisms, they have played an important role in traditional medicine with diverse pharmacological activities, including antioxidant, anti-inflammatory, antimicrobial, cardioprotective, and anticancer effects (Ağagündüz et al., 2022). Importantly, they can modulate the composition and diversity of the gut microbiota, promoting beneficial microbial communities that influence the tumor immune microenvironment and enhance antitumor immunity (Liu et al., 2019; Zitvogel et al., 2017; Cao et al., 2024; Shuwen et al., 2020). The overall interplay between natural products, gut microbiota, immune cells, cytokines, and the tumor microenvironment is illustrated in Figure 2.

Natural products regulate gut microbiota through three primary strategies. First, they reshape microbial composition, selectively promoting the proliferation of beneficial taxa such as *Akkermansia* and *Bifidobacterium*, while suppressing harmful bacteria, including *Clostridium* and *Fusobacterium*, thereby enhancing microbial homeostasis (Van Buiten et al., 2024; Lu et al., 2021). Second, they regulate microbial metabolites by modulating microbial metabolic pathways. These metabolites, in turn, modulate host immune responses and improve metabolic and inflammatory balance (Meiners et al., 2025; Che et al., 2024). Third, natural products maintain intestinal barrier integrity by strengthening tight junctions, enhancing mucosal repair, and reducing LPS translocation into circulation, which collectively alleviates systemic inflammation and preserves a favorable intestinal immune microenvironment (Mo et al., 2024; Martins-Gomes et al., 2024).

### 4.1 Natural products reshape microbial composition

Accumulating evidence indicates that restoration of gut microbial ecology is one of the primary mechanisms through which natural products enhance antitumor immunity. These compounds selectively enrich beneficial commensal microorganisms while suppressing opportunistic pathogens, thereby restoring microbial diversity and creating an intestinal ecosystem favorable for immune activation.

Natural polysaccharides are among the most extensively investigated microbiota modulators. Tea polysaccharides (TPS) significantly increased the relative abundance of Bacteroidota while reducing Bacillota at the phylum level, reflecting an improvement in microbial community structure (Yang et al., 2025). Similarly, Pleurotus eryngii polysaccharide (PEP) enriched beneficial bacterial families including Porphyromonadaceae, Rikenellaceae, Bacteroidaceae, and Lactobacillaceae, while reducing Lachnospiraceae and Ruminococcaceae (Ma et al., 2017). Ganoderma lucidum polysaccharides (GLPs) likewise promoted the growth of *Alistipes* and *Parabacteroides* at the genus level, as well as Prevotellaceae and Peptostreptococcaceae at the family level, while suppressing *Oscillospira* at the genus level and Lachnospiraceae and Desulfovibrionaceae at the family level in breast cancer (BC) and CRC mice (Li et al., 2018; Luo et al., 2018).

Polyphenols also regulate the intestinal microbiota through modulation of bacterial growth. For example, the polyphenol castalagin, isolated from berries, was found to enrich bacteria associated with improved immunotherapeutic responses, including Ruminococcaceae and Alistipes, in the gut of mice, thereby increasing the CD8^+^/FOXP3^+^CD4^+^ T cells ratio within the TME (Messaoudene et al., 2022). Similarly, curcumin, a polyphenolic compound from Curcuma longa (turmeric), acts in a prebiotic-like manner to modulate the intestinal microbiota, restoring microbial balance by reducing pathogenic bacteria such as Escherichia-Shigella and increasing beneficial taxa like Lachnoclostridium and Lactobacillaceae, thereby strengthening intestinal barrier integrity (Zam, 2018; Balaji et al., 2025). In addition, Epigallocatechin-3-gallate (EGCG), the principal bioactive polyphenol in green tea, remodeled the gut microbiota by enriching Akkermansia, which was associated with improved responses to PD-1/PD-L1 immunotherapy (Wu et al., 2021).

Comparable microbial remodeling has also been observed following treatment with glycosides, flavonoids, and terpenoids. Ginsenosides, the most crucial active ingredients in ginseng with a broad range of pharmacological effects, are particularly notable for their anticancer effects (Liu and Fan, 2018). They modulate the gut microbiota by increasing the abundance of beneficial bacteria such as Lactobacillus, Oscillibacter, Bacteroidota, Lachnospiraceae, Bifidobacteriaceae, and Akkermansia, while markedly reducing harmful taxa including Helicobacter, Ruminococcaceae, and Bacillota. Consequently, ginsenosides downregulate inflammatory cytokines IL-17, IL-18, IL-22, and IL-6 associated with ILC3 and Th17 cells, thereby regulating ILC3 immune responses and suppressing the JAK-STAT3 signaling pathway in CRC mice (Ai et al., 2024; Bai et al., 2024). Kaempferol, as a kind of flavonoid, is mainly derived from the ginger plant *Kaempferia galanga* L. It regulates intestinal flora by significantly increasing the abundance of beneficial bacteria and inhibiting the growth of potentially pathogenic bacteria (Li et al., 2022). Terpenoids, particularly phytosterols, contribute to gut microbial homeostasis by maintaining microbial diversity and enriching beneficial bacteria such as *Lactobacillus pentosus* (*L. pentosus*). These microbiota changes are accompanied by suppression of the PI3K/Akt signaling pathway and enhanced apoptosis of colon cancer cells (Ma et al., 2019).

In addition to isolated natural compounds, complex herbal formulations also exhibit robust microbiota-remodeling capacity. Jujube powder, fungal spore powder (IHS), Gegen Qinlian decoction (GQD), and Sini decoction (SND) enrich beneficial bacterial genera such as *Lachnospiraceae*, *Bacteroides*, *Bifidobacterium*, and *Lactobacillus*, while reducing disease-associated microorganisms including *Prevotellaceae*, *Mucispirillum*, and *Bacteroides fragilis*, thereby restoring microbial ecological homeostasis in preclinical tumor models (Jing et al., 2021; Yang et al., 2022; Lv et al., 2019; Wang et al., 2021).

### 4.2 Natural products modulate microbial metabolites and immune signaling

Beyond reshaping microbial composition, natural products regulate the production of microbiota-derived metabolites that function as critical mediators linking intestinal microorganisms with host immune responses. These metabolites, particularly SCFAs, bile acids, indole derivatives, and selected alkaloid metabolites, activate multiple signaling pathways that ultimately promote antitumor immunity.

SCFAs are among the best-characterized microbiota-derived metabolites. Several natural products promote SCFA production by enriching fermentative bacteria. For example, bilberry anthocyanins increase the abundance of butyrate-producing bacteria, including Lachnospiraceae, Ruminococcaceae, and Clostridia, leading to elevated butyrate production that preserves intestinal barrier integrity, promotes intratumoral CD8^+^ T-cell infiltration, and enhances the efficacy of α-PD-L1 therapy in murine MC38 colon cancer models (Liu et al., 2020; Wang et al., 2020). Similarly, saponins from Pulsatilla chinensis enhance gut SCFA production, activate GPR43–NLRP3 signaling, and suppress pro-inflammatory cytokine production (Li et al., 2023). In addition, sterols enrich Lactobacillus pentosus and increase fecal SCFA levels in mice, thereby inhibiting PI3K/Akt signaling and inducing apoptosis in colon cancer cells (Ma et al., 2019). Together, these findings suggest that natural products enhance antitumor immunity by promoting SCFA production and activating downstream immune signaling pathways.

In addition to SCFAs, Natural products can also regulate microbiota-derived bile acid metabolism and downstream immune signaling. Kaempferol, a flavonoid compound, suppresses secondary bile acid biosynthesis and modulates NOD-like receptor signaling, thereby contributing to intestinal immune homeostasis and antitumor immunity (Li et al., 2022).

Microbiota-derived indole derivatives represent another important class of immunoregulatory metabolites. Generated from bacterial Trp metabolism, these metabolites regulate immune responses mainly through activation of the AhR. *Icariside I*, a natural flavonoid isolated from Epimedium, promotes the production of indole derivatives and SCFAs, activating AhR and GPR41/GPR43 signaling to enhance the infiltration of CD4^+^ T cells, CD8^+^ T cells, NK cells, and NKT cells (Chen et al., 2021). Likewise, the microbiota-derived alkaloid metabolite trigonelline, produced by *Blautia coccoides*, potentiates anti-PD-1 immunotherapy by promoting CD8^+^ T cell-mediated antitumor immunity (Wang et al., 2024a). Collectively, these findings demonstrate that natural products modulate microbial metabolite production and downstream immune signaling, thereby enhancing antitumor immunity and improving the efficacy of cancer immunotherapy.

### 4.3 Natural products preserve intestinal barrier integrity

Maintenance of intestinal barrier integrity is essential for preventing microbial translocation and sustaining effective antitumor immunity. In addition to remodeling gut microbiota and microbial metabolites, natural products reinforce epithelial barrier function and restore intestinal immune homeostasis (Zhang et al., 2021; Zam, 2018; Balaji et al., 2025; Wu et al., 2021).

Several natural products improve epithelial barrier function by enriching beneficial bacteria. Pectin increases the abundance of Bifidobacteriaceae, Lactobacillaceae, and Ruminococcaceae, thereby promoting intestinal barrier integrity and facilitating CD8^+^ T-cell infiltration into the TME (Zhang et al., 2021). Similarly, curcumin restores microbial balance by reducing pathogenic bacteria while enriching beneficial taxa, leading to improved intestinal barrier function and reduced inflammation (Zam, 2018; Balaji et al., 2025). EGCG also alleviates colonic barrier dysfunction by enriching *Akkermansia* and enhancing butyrate production, which are associated with improved responses to PD-1/PD-L1 immunotherapy (Wu et al., 2021).

Collectively, these findings indicate that natural products preserve intestinal barrier integrity while limiting chronic inflammation, creating a favorable immune environment that enhances the efficacy of cancer immunotherapy. A summary of representative natural products, their microbiota-modulating effects, and associated immunological outcomes is provided in Table 3.

**TABLE 3:** Representative natural products and their functional effects.

Cancer types	Natural products	Active ingredients	Classes	Microecological change	Immune mechanism	Ref.
CRC	Tea	TPS	Polysaccharides	Bacteroidota↑ *Bacillota*↓	Promotes SCFAs synthesis, activates AhR and FXR.	Yang et al., 2025
None	*Pleurotus eryngii*	PEP	Polysaccharides	Porphyromonadaceae↑ Rikenellaceae↑ Bacteroidaceae↑ Lactobacillaceae ↑ Lachnospiraceae↑ Ruminococcaceae ↓	Enhances SCFAs production and improves intestinal barrier function.	Ma et al., 2017
CRC	*Ganoderma lucidum*	GLPs	Polysaccharides	*Alistipes*↑ *Parabacteroides*↑ Prevotellaceae↑ Peptostreptococcaceae↑ *Oscillospira*↑ Lachnospiraceae↑ Desulfovibrionaceae ↓	Modulates macrophage function and enhances CD8^+^ T cell infiltration.	Luo et al., 2018
CRC	*Cichorium glandulosum*	Inulin	Polysaccharides	*Bifidobacterium*↑ *Lactobacillus*↑	Produces SCFAs, inhibits EMT, and suppresses metastasis.	Shoaib et al., 2016
None	*Panax ginseng*	GPs	Polysaccharides	*B. vulgatus*↑ *P. distasonis*↑	Reduces Tregs to enhance anti-PD-1 therapy.	Huang et al., 2022
CRC	Cell walls of plants	Pectin	Polysaccharides	Erysipelotrichaceae↑ Bifidobacteriaceae↑ Lactobacillaceae↑ Ruminococcaceae↑	Stimulates T cell infiltration and enhances anti-PD-1 response.	Zhang et al., 2021
FSA BC	Camu-camu	Castalagin	Polyphenols	Ruminococcaceae↑ *Alistipes*↑ Christensenellaceae↑ *Paraprevotella*↑	Increases the CD8^+^/CD4^+^ T cells ratio in TME.	Messaoudene et al., 2022
None	*Curcuma longa L.*	Curcumin	Polyphenols	*Lachnoclostridium*↑ *Lactobacillaceae*↑	Strengthens intestinal barrier integrity.	Balaji et al., 2025
CRC	Bilberry	Bilberry anthocyanins	Polyphenols	Lachnospiraceae↑ Ruminococcaceae↑ Clostridia↓ L. *johnsonii*↓	Promotes butyrate production and CD8^+^ T cells infiltration.	Liu et al., 2020
CRC	Green tea	EGCG	Polyphenols	*Akkermansia*↑	Restores the intestinal barrier, increases butyrate synthesis, and reduces NF-κB and AP-1 activation.	Wu et al., 2021
Pan-Cancer	Polyphenol-rich foods	Resveratrol	Polyphenols	Beneficial bacteria↑ Pathogenic bacteria↓	Activates macrophages and T cells, increasing cytokine expression.	Luo et al., 2025
CRC	*Coptis chinensis*	BBR	Alkaloids	*Desulfovibrio*↓ *Eubacterium*↓ *Bacteroides*↓	Regulates the Tregs/Th17 cells balance and activates FXR/TGR5.	Cui et al., 2018
BCa	*Blautia coccoides*	Trigonelline	Alkaloids	*B. coccoides↑*	Enhances CD8^+^ T cell-mediated anti-PD-1 efficacy.	Wang et al., 2024a
None	*Pulsatilla chinensis*	Saponins	Glycosides	Beneficial bacteria↑ Pathogenic bacteria↓	Activates GPR43–NLRP3 signaling.	Li et al., 2023
LC	Ginseng	Ginsenosides	Glycosides	*Lactobacillus*↑ *Bacteroides*↑ *Clostridium*↑ *Enterococcus*↑	Enhances SCFA metabolism, improves TME, and systemic immunity.	Ai et al., 2024
CRC HCC	Ginseng	Ginsenoside Rk3	Glycosides	Bacteroidota↑ Lachnospiraceae↑ Bifidobacteriaceae↑ *Akkermansia*↑ Firmicutes↓ *Helicobacter*↓	Downregulates IL-17, IL-18, IL-22, IL-6, modulates ILC3 and Th17, suppressing the JAK-STAT3 signaling pathway.	Bai et al., 2024
CRC	*Kaempferia galanga L*	Kaempferol	Flavonoids	Beneficial bacteria↑ Pathogenic bacteria↓	Regulates BAs signaling, increasing the activity of GPCRs.	Li et al., 2022
MM	*Epimedium*	Icariside I	Flavonoids	*Lactobacillus*↑ *Bifidobacterium*↑	Increases CD4^+^ T cells, CD8^+^ T cells, NK cells, and NKT cells.	Chen et al., 2021
CRC	Plants	Sterols	Terpenoids	*L. pentosus*↑	Enhances SCFAs and induces tumor apoptosis via PI3K/Akt inhibition.	Ma et al., 2019
MM	*Dioscorea*	Diosgenin	Terpenoids	*Lactobacillus*↑ *Sutterella*↑ *Bacteroides*↓	Enhances CD4^+^/CD8^+^ T-cell infiltration and IFN-γ expression.	Dong et al., 2018
CRC	Jujube	JP	Others	Clostridiales↑ Ruminococcaceae↑ Lachnospiraceae↑	Increases SCFAs production, CD8^+^ T cell infiltration, and improves anti-PD-L1 response.	Jing et al., 2021
CRC	*I. hispidus*	IHS	Others	*Oscillospira*↑ *Odoribacter*↑ *Rikenella*↑	Promotes CD8^+^ T cell infiltration, regulates JAK/STAT.	Yang et al., 2022
CRC	GOD	Baicalin, baicalein, BBR, puerarin	TCM	*B*. *acidifaciens*↑	Increases CD8^+^ T cells, IFN-γ, and IL-2.	Lv et al., 2019
CRC	SND	Aconitine, liquiritin, isoliquiritigenin.	TCM	*Bifidobacterium*↑ *Lactobacillus*↑ *B. coagulans*↑	Upregulates CD8^+^ T cells and Occludin, suppresses CD4^+^ T cells.	Wang et al., 2021
CRC	CHAC soup	Quercetin, Kaempferol, β-sitosterol	TCM	*Turicibacter↑* *Faecalibaculum*↑ *Lactobacillus*↑	Increases Th17s, CD8^+^ T cells, and NK cells.	Nie et al., 2022

### 4.4 Limitations of natural products-based interventions

Natural products have long contributed to therapeutic discovery, with numerous natural product-derived compounds progressing into clinical development or approved medicines. However, microbiota-targeted natural-product interventions for cancer immunotherapy remain largely supported by preclinical evidence, and their clinical translation requires further validation (Butler et al., 2026). To date, clinical evidence remains limited. Small-scale human studies have shown that dietary pectin, curcumin, and berberine can modulate gut microbial composition, while translational studies of castalagin have associated enrichment of Ruminococcaceae with improved responses to anti-PD-1 immunotherapy (Zhang et al., 2021; Messaoudene et al., 2022). However, these studies have mainly focused on microbiota compositional changes rather than demonstrating direct therapeutic benefits, and prospective randomized clinical trials evaluating microbiota-targeted natural products as adjuvants to cancer immunotherapy are still lacking.

In addition to the limited clinical evidence, several challenges hinder the clinical translation of natural-product-based interventions. Many natural products exhibit poor bioavailability because of limited solubility, low intestinal absorption, rapid metabolism, and extensive first-pass elimination, reducing their therapeutic efficacy in vivo. Furthermore, variability in botanical sources, extraction procedures, purification methods, and formulations leads to inconsistent chemical composition and biological activity. The optimal dosage, treatment duration, administration route, and long-term safety also remain to be established. Moreover, the pharmacokinetic and pharmacodynamic characteristics of natural products, particularly their bidirectional interactions with the gut microbiota, require further investigation (Elkrief et al., 2025).

The major challenges and future perspectives toward their clinical translation are summarized in Table 4. Addressing these challenges will be essential for translating microbiota-mediated mechanisms of natural products into clinically applicable cancer immunotherapy strategies.

**TABLE 4:** Current challenges and future perspectives for natural product-mediated microbiota modulation in cancer immunotherapy.

Challenges	Current knowledge gaps	Future research priorities
Limited clinical evidence	Most findings are from preclinical studies	Conduct well-designed clinical trials
Microbiota variability among individuals	Lack of universal microbial biomarkers	Develop personalized microbiome-based strategies
Unclear microbiota–metabolite–immune interactions	Mechanisms remain incompletely understood	Integrate multi-omics approaches
Translational barriers of natural products	Poor bioavailability and unclear pharmacological properties	Develop optimized formulations and delivery systems
Emerging role of intratumoral microbiota	Contribution of tumor-resident microbes to immunotherapy remains unclear	Explore interactions between gut and intratumoral microbiota

## 5 Discussion

Recent advances have established natural products as important regulators of the gut microbiota–immune axis in cancer immunotherapy. By reshaping microbial composition, regulating microbiota-derived metabolites, and maintaining intestinal immune homeostasis, natural products can influence the tumor immune microenvironment and enhance antitumor responses. Despite the promising potential of microbiota-based strategies, several challenges remain for clinical translation. The high interindividual variability of gut microbiota, incomplete understanding of causal relationships between microbial alterations and immune responses, and lack of standardized approaches represent major obstacles. Moreover, clinical evidence supporting natural product-mediated microbiota modulation in cancer immunotherapy remains limited, highlighting the need for further clinical validation and biomarker-guided personalized strategies.

Future research should focus on deciphering the functional interactions among natural products, microbial communities, microbial metabolites, and host immunity rather than solely characterizing changes in microbial composition. Integrative approaches combining metagenomics, metabolomics, transcriptomics, spatial transcriptomics, and single-cell sequencing, together with emerging technologies such as microbiome engineering, synthetic biology, and artificial intelligence-assisted approaches, will facilitate the identification of key microbial taxa, immunomodulatory metabolites, and host signaling networks that determine therapeutic responses. These advances may enable precise manipulation of microbial ecosystems, discovery of reliable biomarkers, and development of personalized microbiota-targeted strategies. Moreover, the recent identification of intratumoral microbiota highlights the importance of considering microbial ecosystems beyond the gut, as tumor-resident microorganisms can regulate local immune responses, influence drug metabolism, and modulate responses to immunotherapy (Lu et al., 2024; Dai et al., 2024; Nejman et al., 2020; Bao and Zhang, 2024). Considering both gut and intratumoral microbiota may provide a more comprehensive understanding of tumor–microbiota–immune interactions and facilitate the development of next-generation combination therapies.
